# A new approach to endoscopic DCR

**DOI:** 10.5935/1808-8694.20120002

**Published:** 2015-11-20

**Authors:** Amit Pal Singh, Vineet Narula, Ravi Meher

**Affiliations:** a M.S (E.N.T), D.N.B (Senior Resident Maulana Azad Medical College Delhi India); b M.S (ENT) (Senior Resident Maulana Azad Medical College Delhi India); c Dr. M.s E.n.t (Associate Professor Maulana Azad Medical College Delhi India)

**Keywords:** dacryocystorhinostomy, endoscopy, nose

## Abstract

**Aim:**

To compare a composite technique of Endoscopic Dacrocystorhinostomy with the conventional technique.

**Methods:**

A randomised prospective study was carried in the department of Otolaryngology Maulana Azad Medical College. Thirty patient selected for Endoscopic DCR were divided into two groups, one of which underwent conventional Endoscopic DCR and the other group were treated with a newer technique using cautery, cold instrumentation and laser at different steps of Endoscopic DCR. The patients were followed up for Nine months.

**Results/Conclusion:**

By using cautery, cold instrumentation and laser at different steps of Endoscopic DCR we were able to achieve a success rate of around 94% with this composite technique as compared to 83.3% in conventional Endoscopic DCR surgery.

## INTRODUCTION

Epiphora or abnormal tearing occurs because of blockage in the lacrimal drainage system, which impairs normal tear channelling into the nose. As a result of the stagnation recurrent infection may also occur. The dacryocystorhinostomy operation, which involves fistulization of the lacrimal sac into the nasal cavity, may alleviate the symptoms. It was first described via an external approach by Toti in 1904^1^. The first intranasal DCR was described by Caldwell in 1893^2^. In 1989, McDonogh & Meiring^3^ described the endoscopic transnasal DCR. Since this description, a number of modifications using lasers have also been described as a useful tool in endoscopic DCR. Modifications have been described using the Holmium: YAG, argon, carbon dioxide and KTP laser^2^^,^^4^^,^^5^. A transcanalicular approach with the Neodymium: YAG laser has also been described^6^. All these techniques have their advantages and disadvantages. We in this study have tried to use the best of each technique to come up with a composite technique for endoscopic DCR, using cautery, cold instrumentation and laser at various steps, which in our prospective would give better results with least number of complications for the patient.

## METHOD

Patients diagnosed as having nasolacrimal duct obstruction were included in this study. All patients were assessed by an ophthalmologist and had repeated sac washouts, which failed to improve their symptoms. Patients excluded from the study were those who had evidence of presaccal canalicular obstruction or associated sinonasal disease like polyps. Once the diagnosis was made, patients were referred to the ENT department for assessment and pre-operative counseling. Those who consented for surgery were then listed for an Endoscopic DCR.

Thirty such patients who underwent endonasal endoscopic DCR at our institute from January 2008 to January 2009 were included in this study. These patients were randomly divided in two groups by taking every alternate patient in one group, 15 patients in the first group underwent a conventional endoscopic DCR while in the latter group 15 patients were operated using the composite technique developed by us. The same surgeon (SM) performed the procedure under a local anaesthetic in all cases.

### Conventional technique

The technique used was similar to that described by McDonogh and Meiring^3^. Key aspects of the technique are as follows. The middle meatus was prepared using 1:2 lakh lignocaine: adrenaline. A 0° Storz Hopkins rodlens endoscope was used to visualise the middle meatus. A lachrymal dilator was used to dilate the punctum of the superior canaliculus. A Bowmann lachrymal probe was used to assess patency of the superior, inferior and common canaliculus. A Rosen tympanoplasty knife was used to elevate the mucosa and the underlying lacrimal bone off the lacrimal sac. This was then removed with a Blakesley forceps. The thick frontal process of the maxilla overlying the anterior portion of the lacrimal sac was trimmed using a fine backward biting Hyack-Kofler punch. The entire medial wall of the lacrimal sac, thus, was exposed, with the endolight illuminating and tenting its medial wall. This was incised in its vertical length as anteriorly as possible. A posteriorly based flap of the medial sac wall was then created and removed with the Blakesley forceps. This exposed the internal structure and contents of the lacrimal sac to the middle meatus.

Only sufficient nasal mucosa (overlying the sac) was removed to expose the sac, taking care that once bone over the sac had been removed the mucosa edge and lacrimal sac edge were closely opposed. This kept the residual exposed bone around the sac to a minimum. Care was also taken to remove the mucosa, bone and medial sac wall inferiorly so as to prevent a sump effect of the draining tears. No stents were used and packing was done. The patient was discharged the following day postoperatively.

### Composite technique

The initial part of the procedure was similar to the conventional technique. The middle meatus was prepared using 1:2 lakh lignocaine: adrenaline. A 0^0^ Storz Hopkins rodlens endoscope was used to visualise the middle meatus. A lachrymal dilator was used to dilate the punctum of the superior canaliculus. A Bowmann lachrymal probe was used to assess patency of the superior, inferior and common canaliculus. The mucosal region over the maxillary line was marked with cautery including area of 1.5 cm in diameter starting from upper attachment of middle turbinate till the inferior turbinate inferiorly. This area was then cauterised completely and the charred tissue was removed exposing the bony part below. The thick frontal process of the maxilla overlying the anterior portion of the lacrimal sac was trimmed using a fine backward biting Hyack-Kofler punch. The entire medial wall of the lacrimal sac, thus, was exposed, with the endolight illuminating and tenting its medial wall.

Then a canalicular probe was introduced through the inferior cannula and a diode laser probe was passed through it. Once the illumination of the probe was seen in the middle of the lacrimal sac intranasal, the laser was fired creating an ostium in the medial wall of the lacrimal sac. The ostium was widened enough to prevent any sum developing inferiorly. No stents were used and packing was done. The patient was discharged the following day postoperatively.

### Postoperative care and follow up

Post-operatively, patients were followed up within one month, at three months and, finally, at 9 to 12 months. Lacrimal syringing was performed daily in the first three days postoperatively and subsequently alternate days in the first week and weekly thereafter for the first month. The patient's relief of symptoms and endoscopic visualisation of a middle meatal ostium into the lacrimal sac measured success.

## RESULTS

Out of the Thirty patients who were selected 20 were female (66.6%) and 10 were male (33.3%) between the age of 15 years to 60 years (mean = 47.9 years). These patients had presented to the outpatient department with epiphora of duration of 3 months to 6 year (mean = 3.2 years). Chronic dacryocystitis was of right side in 14 (46.6%) and of left side in 16 (53.3%) of these patients. Out of these thirty patients, 4 (13.3%) patients had sufficient amount of deviated septum which warranted them to undergo septoplasty.

Thirty patients who were planned for Dacryocystorhinostomy (DCR) procedure were divided into two group. 15 underwent surgery by conventional method described above while the other 15 underwent surgery by the composite technique described above. The same surgeon, to eliminate any operative bias carried all surgeries. The two techniques were compared by observing the number of recurrent cases, postoperative adhesion seen during routine endoscopic examination at 3 months and 6 months, number of cases with severe intraoperative bleeding resulting in abandoning of the procedure and number of cases with postoperative bleeding (Table 1).

**Table 1:** Comparison between the two techniques.

	Composite technique	Conventiona technique
Demographic Distribution	15 (8 female,7 male)	15 (10 female, 5 male)
Operative side	8 right,7 left	12 right, 3 left
Recurrence	1 (6.6%)	4 (26.6%)
Adhesions	2 (13.3%)	6 (40%)
Intraoperative bleed	0	1 (6.6%)
Postoperative bleed	0	1 (6.6%)

Out of the 15 who underwent conventional endoDCR eight were female and seven male. Right eye was operated in 53.3% of the patients selected and the left eye was operated in 46.6% patient selected. Out of the 15 patients who underwent DCR through conventional technique, four (26.6%) patients had recurrence of the disease within 6 months of follow up. On endoscopic examination, six out of the 15 patients so selected had adhesions in the region of the middle meatus between the middle turbinate and the lateral nasal wall or between thee septum and the lateral nasal wall. Only in one patient, intraoperative bleed severe enough to cause the procedure to be abounded was seen. 1 patient had bleeding post operatively which required a repacking to be done.

Out of the 15 in whom we performed the composite technique so described, 10 were female and five male. Right eye was operated in 80% of the patients selected and the left eye was operated in 20% patient selected. Out of the 15 patients who underwent DCR through composite technique, only one (6.6%) patient had recurrence of the disease within 6 months of follow up. On regular examination with endoscope, only two out of the 15 patients so selected had adhesions in the region of the middle meatus that to between the septum and the lateral nasal wall. None of the patients who underwent DCR by the composite technique had any episode of intraoperative or postoperative bleeding. Our analysis is based on the percentage rate of occurrence in parameters taken by us (recurrence, adhesions, intraoperative bleed and post operative bleed) in both the techniques.

## DISCUSSION

Most of the studies conducted of Endoscopic DCR have a recorded a success rate of around 80%^7^, ^8^, ^9^, ^10^ which is lower than External DCR success rate of which are around 90%^11^, ^12^, ^13^, ^14^. However, with all the advantages Endoscopic DCR provides it is essential to develop a technique which could increase the success rate of this surgery. To achieve this we developed a composite technique, which utilizes the best available method at each step of Endo DCR, to increase the success rates, which could match or surpass External DCR.

We divided Endo DCR into three main steps. Step 1 was removing the mucosa in the area of the lacrimal sac. Step 2 was to remove the bone overlying the sac. Step 3 incising the medial wall of the lacrimal sac.

A number of studies have been conducted whether to preserve or not to preserve the mucosal flap of the lateral nasal wall over the lacrimal sac area. Ramakrishana et al.^15^ performed 27 Endoscopic DCR's in 20 patients between May 2003 and October 2006 without mucosal flap preservation. They noticed a success rate of 100% for anatomic patency and 93% for complete resolution of epiphora. They concluded that mucosal preservation is not essential to achieve a good success rate. We agree with this study but to remove the mucosa usually a cold instrument like a 45 degree upturn Beksely's forceps is used. Using such instrument inevitably leaves mucosal tags which can cause adhesions resulting in failure but by using a radiofrequency cautery to burn the mucosa overlying the lacrimal area without leaving any mucosal tags behind we were able to decrease adhesion formation, only in 13.3% of our cases as comparable to those of the conventional technique (40%). We were also able to achieve a much more dry field, which is essential for the success of any surgery (there were no cases of intraoperative bleed in our study).

To achieve a high success rate in Endoscopic DCR it is essential to remove the bone overlying the lacrimal sac until the entire medial wall and most of anterior wall of the lacrimal sac is visible^16^. This can not be achieved by using a laser probe and is the cause of high failure rate in case of laser DCR. However, by using a Kerrison punch one can achieve this with ease just like in our technique, helping us to achieve a high success rate.

Welham & Wulc^17^ found that the problems with the size and location of the internal ostia were the cause of failure in 52% of cases. In their study they found that placement of the ostium too close to the middle turbinate results in adhesions and stenosis resulting in failure and the larger size of the ostium is important to have a successful surgery. Therefore, it is essential to remove a large part of the medial wall of the sac. While this can be done by just incising the medial wall of the sac by a sickle knife but this can cause inadvertent damage to the lateral wall of the sac, there can be inadequate removal of the wall decreasing the ostium size, and tags can be left behind resulting in restenosis of the ostium with greater chances of failure. To overcome this we used a Diode laser to burn our way through the medial wall of the lacrimal sac in a controlled manner. This not only helped us to create an ostium of the size and the site required, which were essential for a successful surgery but also prevented any inadvertent damage of the lateral wall of the sac. There were also no mucosal tags left behind to cause stenosis of the ostium resulting in failure.

Lee & Yen^18^ in 2011 conducted a meta analysis of all the recent studies on laser DCR and found that average success rate for laser DCR was 83.73% with a decreased intraoperative bleeding and significant operative time. Ozcimen et al.^19^ in 2010 conducted a study on 60 patients performing endoscopic laser DCR and found the success rate to be 83.3%. Drnovsek-Olup & Beltram^20^ in 2010 conducted a study on 126 patients performing endoscopic laser DCR and found the success rate to be 83.3%. It is clear from the above mentioned studies that laser DCR alone has a low success rate and so our composite technique increases the success rate by taking advantage of all the techniques (cautery, cold instrumentation and laser) at each step of the surgery.

In this era of changing trends we have tried to develop a technique which would help us to achieve higher success rate with least amount of morbidity. In this study, we have tried to prove the same. We know that the sample size is small hence a larger sample size has to be taken and a larger study needs to be done. Nevertheless, the initials trends are very encouraging and this technique can change the way endoscopic DCR can be done.

## CONCLUSION

By using cautery, cold instrumentation and laser at different steps of Endoscopic DCR we were able to achieve a success rate of around 94% with this composite technique as compared to 83.3% in conventional Endoscopic DCR surgery. These rates were not only higher than the conventional technique but also matched the success rate of External DCR surgery with all the advantages Endoscopic DCR can provide (i.e. lack of skin scar). However our sample size is small and further research work need to be carried out but whatever results we observed, it is our belief that this technique can be used to increase the success rate of Endoscopic surgery and to minimise the complications faced by the patients.
